# Association between Hyperuricemia and Metabolic Syndrome: An Epidemiological Study of a Labor Force Population in Taiwan

**DOI:** 10.1155/2015/369179

**Published:** 2015-07-26

**Authors:** Cheng-Yu Wei, Chia-Cheng Sun, James Cheng-Chung Wei, Hsu-Chih Tai, Chien-An Sun, Chian-Fang Chung, Yu-Ching Chou, Pi-Li Lin, Tsan Yang

**Affiliations:** ^1^Department of Neurology, Show Chwan Memorial Hospital, Changhua County 50008, Taiwan; ^2^Department of Neurology, Chang Bing Show Chwan Memorial Hospital, Changhua County 50544, Taiwan; ^3^Department of Exercise and Health Promotion, College of Education, Chinese Culture University, Taipei City 11114, Taiwan; ^4^Physical Examination Center, Show Chwan Memorial Hospital, Changhua County 50008, Taiwan; ^5^Division of Allergy, Immunology and Rheumatology, Chung Shan Medical University, Taichung City 40201, Taiwan; ^6^Department of Public Health, College of Medicine, Fu-Jen Catholic University, New Taipei City 24205, Taiwan; ^7^Sleep Center, Chang Bing Show-Chwan Memorial Hospital, Changhua County 50544, Taiwan; ^8^School of Public Health, National Defense Medical Center, Taipei City 11490, Taiwan; ^9^Department of Nursing, Meiho University, Pingtung County 91202, Taiwan; ^10^Department of Health Business Administration, Meiho University, Pingtung County 91202, Taiwan

## Abstract

The increasing prevalence of metabolic syndrome (MetS) has become an important issue worldwide. Metabolic comorbidities of hypertension, obesity, and hyperlipidemia are shown as important risk factors for incident gout. The purpose of this study was to investigate the relationship between hyperuricemia and MetS. This is a cross-sectional study. The effective sample included 21,544 individuals who received worker health examinations at a local teaching hospital in Changhua County from 2008~2012. We used multiple logistic regression analysis to investigate the influences of hyperuricemia on MetS. The results showed that individuals with MetS had significantly higher blood pressure, fasting plasma glucose, triglycerides, waist circumference, and high-density lipoprotein cholesterol than those without MetS (*P* < 0.001). Multiple logistic regression analysis revealed hyperuricemia to be an important factor of MetS. The risk of developing MetS is higher with high levels of serum uric acid (SUA) and the odds ratio (OR) of having MetS is 4.98 times higher for Tertile 3 than for Tertile 1 (95% CI = 4.16–5.97) and 4 times higher for Quartile 4 than for Quartile 1 (95% CI = 3.59–4.46). In conclusion, males are more likely to develop MetS than females, and the risk of having MetS increases with age and SUA concentration.

## 1. Introduction

Gouty arthritis is a common type of chronic arthritis. Hyperuricemia and crystallization of monosodium urate in joints are considered as the important risk factors of gouty arthritis [1]. The relations between hyperuricemia and comorbidities, for instance, hypertension, obesity, and MetS, have been demonstrated in many epidemiologic studies [2, 3].

Previous report suggested the high prevalence of MetS in gouty patients, and also it defined the association between insulin resistance and gouty arthritis [4]. Recent studies showed that the prevalence of MetS was high among patients with gout, 44% in Korean men and 82% in Mexican men compared with 5% in the Korean general population [5–7]. Therefore, it is highly suggested to diagnose and treat gout as one of the metabolic diseases [5, 8]. Gout is not considered as a simple joint disease and is associated with obesity, cardiovascular disease, hypertension, and MetS with increased mortality [9–11].

There is a tendency for younger individuals to have MetS. Also, MetS is closely related to the occurrence and mortality of chronic conditions such as cardiovascular diseases (CVD), cardiac diseases, diabetes mellitus (DM), and hypertension [12–14].

Epidemiological studies on MetS in different countries and regions show drastically different results. The primary reasons for such drastic differences can be the different characteristics of the participants (e.g., age, ethnicity, socioeconomic status, and abnormal risk factors), the study periods, or different diagnostic definitions. In the United States, the prevalence of MetS is estimated to be 27% (25.2% in men and 29% in women) [15]; and in Taiwan, it is 15.7% (18.3% in men and 13.6% in women) [16].

Previous epidemiological surveys showed that the hyperuricemia is related to the prevalence of MetS [17, 18]. However, previous studies, for the most part, recruited the general public as research participants and few studies investigated worker populations. Therefore, the purpose of this study was to investigate the relationship between hyperuricemia and MetS in a labor force population. Thus, identifying asymptomatic individuals with a high risk of developing MetS may lead to improvements in prevention and treatment of the condition and of subsequent cardiovascular events and gouty arthritis.

## 2. Methods

We conducted a cross-sectional study on individuals that had undergone worker health screening at a local teaching hospital in Changhua County from 2008~2012. The physical examination document includes physical examination and biochemical blood tests. All individual's examination collecting progress and analysis progress were in accordance with the hospital standard operating procedures, and the laboratory analysis was in accordance with the quality assurance (QA) and quality control (QC). The total sample size was 33,776 with a valid sample size of 21,544 after excluding those with incomplete data on physical examination and biochemical blood tests.

Definition of terms is as follows. (I) In order to understand the current international classification of SUA as criteria of elevated SUA, the present study classified serum SUA levels into (A) SUA > 7.0 mg/dL for males and SUA > 6.0 mg/dL for females [19, 20]; (B) three subgroups in light of the concentration of SUA: (a) SUA < 7 mg/dL, (b) 7 mg/dL ≦ SUA < 9 mg/dL, and (c) SUA ≧ 9 mg/dL [21]; (C) four subgroups among males: (a) SUA < 5 mg/dL, (b) 5 mg/dL ≦ SUA < 6 mg/dL, (c) 6 mg/dL ≦ SUA < 7 mg/dL, and (d) SUA ≧ 7 mg/dL; and four subgroups among females: (a) SUA < 4 mg/dL, (b) 4 mg/dL ≦ SUA < 5 mg/dL, (c) 5 mg/dL ≦ SUA < 6 mg/dL, and (d) SUA ≧ 6 mg/dL [22]. (II) MetS status was defined according to the criteria set in the Third Report of the National Cholesterol Education Program (NCEP) Expert Panel on Detection, Evaluation, and Treatment of High Blood Cholesterol in Adults (Adult Treatment Panel III, ATP-III) [23]. We utilized previously established modifications for Asian populations, using waist circumference (WC) cut-off points [24]. Any three of the following five criteria were grounds for identifying MetS: (a) abdominal obesity: WC: ≧90 cm in men and ≧80 cm in women; (b) raised triglyceride (TG): ≧150 mg/dL; (c) reduced high-density lipoprotein cholesterol (HDL-C): <40 mg/dL in men and <50 mg/dL in women; (d) raised blood pressure (BP): BP of at least 130/85 mmHg or taking antihypertensive medication; and (e) raised fasting plasma glucose (FPG): ≧110 mg/dL and/or taking antiglycemic medication.

The physical examination comprised BP and anthropometric measurements, including height, weight, and body mass index (BMI). Height was measured to the nearest 0.1 cm, without shoes, using a stadiometer. Weight was measured in light clothing, without shoes, using a beam balance scale, and was recorded to the nearest 0.1 kg. BMI was calculated as weight (kg) divided by height^2^ (m^2^). Well-trained nurses measured systolic BP (SBP) and diastolic BP (DBP) two times in the left arm of seated participants, according to a standardized protocol. A third BP measurement was made if the first two BP readings differed by more than 10 mm Hg. The average of the two closest readings was calculated to determine the reported BP for each participant. The items in the blood examination included TG, SUA, FPG, and HDL-C. The sample was venous blood drawn after 8 hours of fasting and was sent to the lab within an hour and analyzed by a Hitachi-7070 biochemical analyzer.

Data in the present study were analyzed using SPSS 17.0 (SPSS for Windows release 17.0), with a significance level of *α* = 0.05. Descriptive statistics used the average value, the standard deviation, the frequency distribution, and the percentage criterion. Inferential statistics used the Chi-square and multiple logistic regression models for analysis.

## 3. Results

Study participants included 21,544 individuals aged over 21 who were receiving a worker health examination in Changhua County. The mean age was 38.4 ± 9.5, with 4,881 individuals (27.0%) in the range of 21–30 years old, 8,940 (41.5%) individuals in the range of 31–40 years old, 4,780 (22.2%) individuals in the range of 41–50 years old, and 2,943 (13.7%) individuals over 51 years old. The majority of the participants were male, with 13,009 males (60.4%) and 8,535 females (39.6%).

Demographic characteristics such as age and gender differed significantly between individuals with and without MetS (*P* < 0.001). Individuals at an older age had high prevalence of MetS, and males had higher prevalence of MetS than females (15.8% versus 6.7%). There were significantly higher ratios of abnormal BP, FPG, TG, WC, and HDL-C in individuals with MetS than in those without MetS; the ratios were 28.0%, 60.5%, 44.7%, 37.8%, and 49.8%, respectively (Table 1).

The ratio of hyperuricemia in individuals with MetS was 24.7%, which is significantly higher than the 8.3% in individuals without MetS (*P* < 0.001). After dividing the SUA concentration into 3 groups, we found that Tertile 3 had an abnormally high ratio of SUA compared to Tertiles 2 and 1 (35.3% versus 22.9% and 8.4%). In addition, we divided SUA concentration into 4 groups, and we found that Quartile 4 had a significantly and abnormally high ratio of SUA compared to Quartile 1 (24.0% versus 5.6%) (*P* < 0.001) (Table 2).

In this study, we added variables that were statistically significant by univariate analysis (i.e., age, gender, and SUA) into multiple logistic regression analysis. The results (Table 3) show that SUA was a significant contributing factor to MetS in multiple logistic regression models I, II, and III. For model I, the OR of individuals with hyperuricemia having MetS was 3.08 times that of individuals with normal SUA (95% CI = 2.82–3.37). With increased age, the ORs of having MetS increased accordingly. For example, the OR of individuals over 51 years old having MetS was 4.46 times that of individuals 21–30 years old (95% CI = 3.81–5.23), and the OR of males having MetS was 1.80 times that of females (95% CI = 1.63–2.00). These ORs are all statistically significant (*P* < 0.001). For model II, we divided the SUA concentration into 3 groups and found that a higher SUA concentration was associated with a higher risk of MetS. For those individuals with SUA ≧ 9 mg/dL, the risk of having MetS was 4.98 times higher (95% CI = 4.16–5.97). With increased age, the odds of MetS increased 2.24~4.59 times (ORs = 2.24~4.59). Males had 1.59 times higher OR of MetS than females (95% CI = 1.42–1.77). In addition, in model III, we divided the SUA concentration into 4 groups and found that a higher SUA concentration was associated with a higher risk of MetS. Individuals with a SUA concentration in the Quartile 3 and Quartile 4 groups had 1.70~4.00 times ORs of MetS, and the ORs associated with increased age were 2.25~4.48 times. As for gender as a risk factor, males had a risk of developing MetS that was 1.65 times (95% CI = 1.49–1.83) higher, and significantly so, than that of females (*P* < 0.001).

## 4. Discussion

Studies show that hyperuricemia not only can cause gout, but also is a high risk factor for atherosclerotic diseases such as CVD and carotid atherosclerosis [25], hypertension [26], DM [27], MetS [28], and cardiac diseases and chronic diseases such as cerebral vascular diseases. Further analysis showed that elevated SUA is associated with a higher risk of developing CVD and mortality as a result of CVD [29]. Our study shows that MetS is associated with age, and increased age is associated with a higher rate of MetS. We observed that individuals over 51 years old had the prevalence rate of 20.9% of developing MetS. Our result is consistent with many previous studies that show that the prevalence of MetS increases with increased age [30]. We also found that males have a higher rate of MetS than females (15.8% versus 6.7%), a result similar to other studies. For example, Balkau et al. (2003) found that prevalence rates of MetS in males and females among 4,293 individuals in the 2001 French National Cholesterol Education Program were 10% and 7%, respectively [31]. Hu et al. (2004) found that among 11,512 Europeans aged from 30 to 89 who did not have DM males had higher prevalence of MetS than females (15.7% versus 14.2%) [32]. Chuang et al. (2004) found that among those receiving health examinations in Taipei City males had higher prevalence of MetS than females (15.5% versus 10.5%). The prevalence rates were 15.7% versus 12.0% after correcting for variables [33].

MetS is commonly accompanied with hypertension, type II DM, hyperlipidemia, stroke, or CVD. MetS is a cluster of three diseases—hypertension, hyperglycemia, and gout. At present, its etiological mechanisms are yet to be understood. But it is clear that MetS is associated with obesity, insulin resistance, and abnormal blood lipids that lead to the above-described three diseases [34]. From our study, it is obvious that individuals with MetS have a significantly higher ratio of abnormal BP, FPG, TG, WC, and HDL-C than those without MetS.

To understand the effects of SUA concentration on MetS, we used multiple logistic regression and conducted analyses using three models, I, II, and III, based on SUA concentration. Variables that showed statistically significant differences in univariate analysis (i.e., age, gender, and SUA) were used in regression models. For different regression models, after controlling other variables, the results showed that the OR of individuals with abnormal SUA having MetS was 3.08 times (95% CI = 2.82–3.37) that of individuals with normal SUA in model I. In models II and III, the ORs of having MetS were higher in those with an elevated SUA concentration, with ORs of 2.65–4.98 and 1.70–4.00, respectively.

A large-scale epidemiological study reported that elevated SUA concentrations are associated with an increased mortality rate; further analyses showed that hyperuricemia seems to be related to hypertension, obesity, MetS, renal diseases, and CVD [35]. A recent study supported the role of SUA itself as an independent risk factor for CVD [26]. Previous studies reached similar conclusions that SUA is significantly associated with cardiovascular risk factors such as hypertension [36], MetS [37], and insulin resistance [38]. Other studies also found that high SUA concentrations are associated with an increased risk of MetS [39]. Follow-up analysis of American residents with hyperuricemia found that males with a SUA value ≧6.5 mg/dL had a 1.6 times higher risk of developing MetS than males with a value <5.5 mg/dL, and females with a SUA value ≧4.6 mg/dL had a 2 times greater risk of developing MetS.

Although the relationship between hyperuricemia and CVD was established in the 19th century, there is still controversy as to whether hyperuricemia plays a role as an indicator or a risk factor [40]. The strong relationship between hyperuricemia and other cardiovascular risk factors such as hypertension, insulin resistance, obesity, and renal insufficiency makes it difficult to establish a causal relationship in epidemiological studies. Recent experiments have made significant contributions in providing important knowledge pertaining to the pathological meaning of hyperuricemia. Symptoms resulting from hyperuricemia can be alleviated by xanthine-oxidase inhibitors or uricosuric agents that reduce SUA concentrations [41]. These results show that lowering SUA can play an important role in preventing CVD. The consensus is that MetS can be considered as a predictive factor for CVD [14]. The traditional view is that impaired renal clearance induced by compensatory hyperinsulinemia can cause hyperuricemia [42]. However, recent studies provide a different perspective, implying that hyperuricemia can be an independent factor predicting the occurrence of MetS and DM, even after controlling for different genders and basic BMI [28].

The findings in previously described studies are similar to those in our study. That is, SUA concentration can be used as an important predictive factor for MetS. The risk of MetS increases with elevated SUA concentrations.

Admittedly, with the cross-sectional nature of this study, it is not possible to establish a causal relationship between hyperuricemia and MetS. This study could not eliminate the potential effects of underlying diseases, medications used, and dietary habits for these diseases among participants on the present findings. It is possible that residual confounding by these factors may also affect the MetS and SUA link. Further population-based prospective studies are needed to elucidate a cause-effect relationship between hyperuricemia and MetS. By using these epidemiologic study findings, it will bring more benefits for health management and prevention of clinical disease.

In conclusion, although hyperuricemia is still not one of the determining factors for MetS, more and more studies are showing that high SUA concentrations are associated with and have negative effects on CVD, insulin resistance, hypertension, and abdominal obesity. Our study also showed that individuals with MetS have higher ratio of having abnormal BP, FPG, TG, WC, and HDL-C than those without MetS. Age, gender, and abnormal SUA were risk factors for MetS as analyzed by the multiple logistic regression models. In addition, a higher SUA concentration is associated with a higher risk of MetS, no matter if the SUA concentrations were divided into three or four groups.

We recommend that dietitians should pay attention to the SUA value in individuals receiving health education for their MetS-related diseases. Related health education should be provided when necessary. As for patients who only have hyperuricemia, dietary health education that is related to MetS should be provided to help the patients control their uric acid value better.

## Figures and Tables

**Table 1:** Correlation analysis of demographic characteristics, biochemistry exam results, and metabolic syndrome (*n* = 21,544).

Variables	No MetS (*n* = 18,927, 87.9%)		MetS (*n* = 2617, 12.1%)		*P* value
	Number	Percentage	Number	Percentage	
Gender					<0.001
Male	10960	84.2	2049	15.8	
Female	7967	93.3	568	6.7	
Age (years)					<0.001
**21–30**	4631	94.9	250	5.1	
31–40	7935	88.8	1005	11.2	
41–50	4034	84.4	746	15.6	
≧51	2327	79.1	753	20.9	
WC (cm)					<0.001
Normal	15327	97.3	431	2.7	
Abnormal (male: ≧90; female: ≧80)	3600	62.2	2186	37.8	
Raised blood pressure (mmHg)^a^					<0.001
Normal	13016	97.6	316	2.4	
Abnormal (≧130/85)	5911	72.0	2301	28.0	
TG (mg/dL)					<0.001
Normal	16227	97.4	436	2.6	
Abnormal (≧150)	2700	55.3	2181	44.7	
Raised FPG (mg/dL)^b^					<0.001
Normal	18524	90.3	1999	9.7	
Abnormal (≧110)	403	39.5	618	60.5	
HDL-C (mg/dL)					<0.001
Normal	17547	93.4	1247	6.6	
Abnormal (*<*40 in men and *<*50 in women)	1380	50.2	1370	49.8	

*Note.* Analyzed by Chi-square test, 2-tailed test, and significance level *α* = .05.

^a^Raised blood pressure ≧130/85 mmHg or currently taking antihypertensive drugs.

^b^Raised fasting plasma glucose ≧110 mg/dL or currently taking oral hypoglycemic agent.

**Table 2:** Correlation analysis of hyperuricemia and metabolic syndrome (*n* = 21,544).

Variable	No MetS (*n* = 18,927, 87.9%)		MetS (*n* = 2617, 12.1%)		*P* value
	Number	Percentage	Number	Percentage	
Hyperuricemia					<0.001
Normal	15074	91.7	1356	8.3	
Abnormal (male > 7; female > 6 mg/dL)	3853	75.3	1261	24.7	
Subgroups of SUA^a^					<0.001
Tertile 1	15190	91.6	1402	8.4	
Tertile 2	3325	77.1	990	22.9	
Tertile 3	412	64.7	225	35.3	
Subgroups of SUA^b^					<0.001
Quartile 1	3274	94.4	194	5.6	
Quartile 2	6007	93.6	411	6.4	
Quartile 3	5406	88.9	673	11.1	
Quartile 4	4240	76.0	1339	24.0	

*Note.* Analyzed by Chi-square test, 2-tailed test, and significance level *α* = .05.

^a^Subgroups of SUA Tertile 1: SUA < 7 mg/dL, Tertile 2: 7 mg/dL ≦ SUA < 9 mg/dL and Tertile 3: SUA ≧ 9 mg/dL.

^b^Subgroups of SUA Quartile 1: male SUA < 5 mg/dL, female SUA < 4 mg/dL; Quartile 2: male 5 mg/dL ≦ SUA < 6 mg/dL, female 4 mg/dL ≦ SUA < 5 mg/dL; Quartile 3: male 6 mg/dL ≦ SUA < 7 mg/dL, female 5 mg/dL ≦ SUA < 6 mg/dL; and Quartile 4: male SUA ≧ 7 mg/dL, female SUA ≧ 6 mg/dL.

**Table 3:** Regression analysis of risk factors of metabolic syndrome.

Item	*β*	Wald	OR (95% CI)	*P* value
Model I				
Gender^a^	0.59	127.37	1.80 (1.63–2.00)	<0.001
Age 2^b^	0.80	117.39	2.23 (1.93–2.58)	<0.001
Age 3^b^	1.21	246.29	3.38 (2.90–3.39)	<0.001
Age 4^b^	1.49	342.42	4.46 (3.81–5.23)	<0.001
Hyperuricemia^c^	1.12	616.32	3.08 (2.82–3.37)	<0.001
Model II				
Gender	0.46	70.50	1.59 (1.42–1.77)	<0.001
Age 2	0.80	118.71	2.24 (1.94–2.59)	<0.001
Age 3	1.22	249.78	3.41 (2.92–3.97)	<0.001
Age 4	1.52	354.88	4.59 (3.92–5.38)	<0.001
Tertile 2^d^	0.98	383.48	2.67 (2.42–2.95)	<0.001
Tertile 3^d^	1.60	305.04	4.98 (4.16–5.97)	<0.001
Model III				
Gender	0.50	90.08	1.65 (1.49–1.83)	<0.001
Age 2	0.81	119.36	2.25 (1.94–2.60)	<0.001
Age 3	1.22	247.95	3.40 (2.92–3.96)	<0.001
Age 4	1.50	344.13	4.48 (3.82–5.25)	<0.001
Quartile 3^e^	0.53	78.90	1.70 (1.51–1.91)	<0.001
Quartile 4^e^	1.38	625.44	4.00 (3.59–4.46)	<0.001

*Note1*. Analyzed by stepwise regression analysis. Considered variables include age, gender, and SUA.

*Note2*. Dependent variable: MetS, 1, with **MetS**; 0, without MetS.

^a^Gender: female = 0, ^b^age: 21–30 = 0.

^c^Abnormal (male > 7; female > 6 mg/dL) = 1; normal = 0.

^d^Subgroups of SUA Tertile 1: SUA < 7 mg/dL, Tertile 2: 7 mg/dL ≦ SUA < 9 mg/dL and Tertile 3: SUA ≧ 9 mg/dL.

^e^subgroups of SUA Quartile 1: male SUA < 5 mg/dL and female SUA < 4 mg/dL.

Quartile 2: male 5 mg/dL ≦ SUA < 6 mg/dL and female 4 mg/dL ≦ SUA < 5 mg/dL.

Quartile 3: male 6 mg/dL ≦ SUA < 7 mg/dL and female 5 mg/dL ≦ SUA < 6 mg/dL.

Quartile 4: male SUA ≧ 7 mg/dL and female SUA ≧ 6 mg/dL.
